# 16S rRNA gene primer choice impacts off-target amplification in human gastrointestinal tract biopsies and microbiome profiling

**DOI:** 10.1038/s41598-023-39575-8

**Published:** 2023-08-03

**Authors:** Tereza Deissová, Martina Zapletalová, Lumír Kunovský, Radek Kroupa, Tomáš Grolich, Zdeněk Kala, Petra Bořilová Linhartová, Jan Lochman

**Affiliations:** 1 Department of Biochemistry, Faculty of Science, Masaryk University, Kamenice 735/5, 62500 Brno, Czech Republic; 2 Department of Gastroenterology and Internal Medicine, University Hospital Brno, and Faculty of Medicine, Masaryk, University, Jihlavská 20, 62500 Brno, Czech Republic; 3 Department of Surgery, University Hospital Brno, and Faculty of Medicine, Masaryk University, Jihlavská 20, 62500 Brno, Czech Republic; 4 Department of Pathophysiology, Faculty of Medicine, Masaryk University, Jihlavská 20, 62500 Brno, Czech Republic; 5 Faculty of Science, RECETOX, Masaryk University, Kotlářská 2, Brno, Czech Republic

**Keywords:** Metagenomics, Oesophageal diseases, Stomach diseases, Microbiome

## Abstract

16S rRNA amplicon sequencing or, more recently, metatranscriptomic analysis are currently the only preferred methods for microbial profiling of samples containing a predominant ratio of human to bacterial DNA. However, due to the off-target amplification of human DNA, current protocols are inadequate for bioptic samples. Here we present an efficient, reliable, and affordable method for the bacteriome analysis of clinical samples human DNA content predominates. We determined the microbiota profile in a total of 40 human biopsies of the esophagus, stomach, and duodenum using 16S rRNA amplicon sequencing with the widely used 515F-806R (V4) primers targeting the V4 region, 68F-338R primers and a modified set of 68F-338R (V1-V2M) primers targeting the V1–V2 region. With the V4 primers, on average 70% of amplicon sequence variants (ASV) mapped to the human genome. On the other hand, this off-target amplification was absent when using the V1–V2M primers. Moreover, the V1–V2M primers provided significantly higher taxonomic richness and reproducibility of analysis compared to the V4 primers. We conclude that the V1–V2M 16S rRNA sequencing method is reliable, cost-effective, and applicable for low-bacterial abundant human samples in medical research.

## Introduction

Over the last decade, investigating the human bacteriome using culture-independent high-throughput sequencing methods has become one of the most frequently used techniques to study bacterial communities inhabiting a wide variety of niches in the human body^1,2^. Access to third-generation technologies coupled with the decreasing costs associated with high-throughput sequencing has resulted in a shift from amplicon 16S rRNA gene sequencing towards sequencing the full 16S rRNA gene and metagenomic/metatranscriptomic sequencing in samples like stools, human vagina^3^ or swabs from the skin or the oral cavity^4^ that contain a predominant ratio of human to bacterial DNA. However, in samples with low concentrations of bacterial DNA or those “contaminated” by host DNA like blood, urine, or human biopsy samples, bacteriome profiling still relies largely on 16S rRNA gene sequencing. Since the amount of data generated is relatively small, it does not require complex bioinformatics analysis^5^, and the price is also more affordable. On the other hand, the results of 16S rRNA amplicon sequencing are critically dependent on the choice of hypervariable sub-regions from the nine available variable regions interspersed throughout the highly conserved 16S rRNA gene sequence as the quality of the information retrieved as well as the taxonomic accuracy can vary significantly depending on the primer set(s) employed^6^. Currently, the vast majority of studies target either the V4 single variable region as in the widely adopted standardized protocol of Earth Microbiome Project (EMP)^7^ or the V1–V3^8^ or V3–V5^9^ variable regions as in the dual-indexing protocol of Human Microbiome Project (HMP). This is mainly because the widely used Illumina sequencing platform produces only short sequences (NextSeq, MiniSeq, iSeq ≤ 300 bases, and MiSeq ≤ 600 bases). Unfortunately, recent studies have shown repeatedly that the commonly targeted 16S rRNA gene sub-region V4 assesses the taxa commonly present in the human body least accurately^6,10,11^. Moreover, together with region V3–V5 it is particularly susceptible to off-target amplification of human DNA^12^, especially in biopsy samples, resulting in the potential loss of rare taxa and bacterial resolution, thus a significant proportion of data goes to waste.

Here we demonstrate a new protocol using a primer set targeting the V1–V2 16S rRNA gene sub-region that drastically decreases off-target amplification of human DNA in biopsy samples from the esophagus, stomach, and duodenum, while significantly increasing alpha diversity and taxonomic accuracy compared to the commonly used primers targeting the V4 region. The amplification primers for the V1–V2 region, including functionalities required for sequencing (flow cell adaptors and indices), were optimized for the Illumina MiniSeq platform with a maximal read length of 150 bp offering a cost-effective option for any laboratory interested in performing high-throughput 16S rRNA gene sequencing. To further increase the performance of taxonomic classifications we included the concatenation of paired-end reads to the bioinformatic pipeline^13^.

## Results and discussion

### The problem of off-target amplification

**Figure 1:**
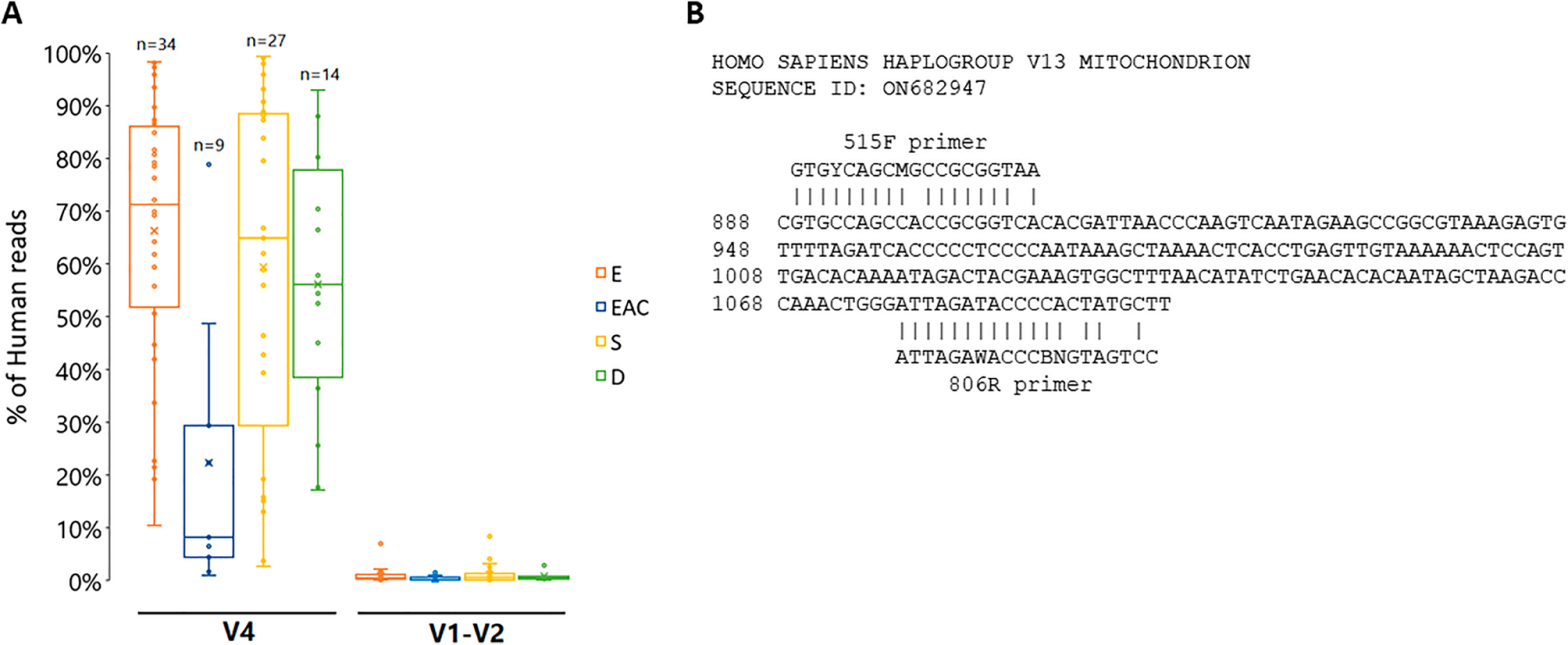
The problem of off-target amplification in samples from esophagus, stomach, and duodenum biopsies. (**A**) Percentage of amplicon sequence variants (ASVs) aligned to the human genome produced by Illumina MiniSeq 2 × 150 bp sequencing of amplicons targeting the V4 and V1–V2 regions. *E* esophagus, *EAC* esophageal adenocarcinoma, *S* stomach, *D* duodenum. (**B**) Alignment of V4 region amplification primers to the *Homo sapiens* mitochondrion haplogroup; sequence ID corresponds to the NCBI nucleotide database.

The widely used standardized protocol for 16S rRNA gene amplicon sequencing^7,14^ turned out to be inadequate due to robust off-target amplification of human DNA during the analysis of bacteriome in samples of different biopsy sites from the upper gastrointestinal (GI) tract. In samples from all three types of biopsy sites (esophagus, stomach, and duodenum), an average of 70% of amplicon sequence variants (ASV) aligned to the human genome, and in some samples it was as high as 98% (Fig. 1A). This resulted in a significant portion of sequencing data from the 16S rRNA gene analysis having to be abandoned due to incorrect taxonomic classification. Interestingly, in the esophageal adenocarcinoma (EAC) samples only about 20% of ASVs aligned to the human genome (Fig. 1) which suggests a different bacterial representation in the tumor environment, as has been seen before^15^. The most prevalent ASV identified according to BLAST was the *Homo sapiens* mitochondrion haplogroup with an E-value of 6e−83 and 100% identity, in which we identified sites with significant alignment to the 515F-806R primer pair used, explaining the observed off-target amplification (Fig. 1B).

This unaddressed problem of significant non-specific amplification has been recently described also during the analysis of breast tissue and esophagus biopsies using primers targeting the V3–V4 region with the standardized protocol for the Illumina MiSeq system^12^. This shows that even though the sequencing of amplified 16S rRNA gene by bacteria-specific primers is an alternative to overcome the common problems related to significant human contamination in amplification-free shotgun metagenomics^4^, the need to use highly degenerate primers may not completely eliminate this problem.

### Taxonomic resolution of a new set of primers eliminating off-target amplification

**Figure 2:**
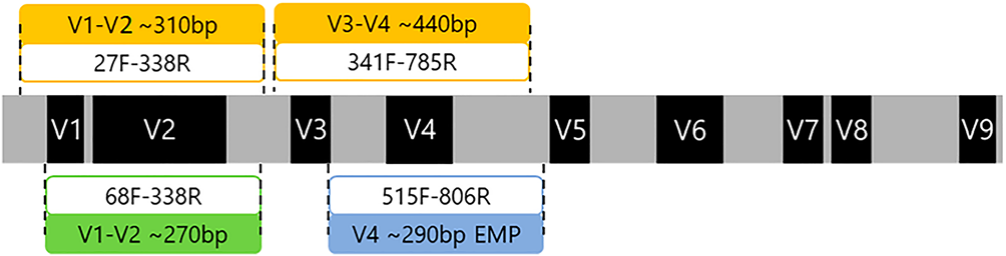
The localization of primers for amplification of the V1–V4 regions of the 16S rRNA gene.

Walker et al*.* demonstrated that human biopsy samples should preferably be amplified using primers targeting the V1–V2 region (S-D-Bact-0027-b-S-20 and S-D-Bact-0338-a-A-18) instead of the V3–V4 region (Fig. 2), as they show lower off-target amplification of human DNA in 16S rRNA gene sequencing^12^. However, they used a two-step amplification protocol for the V1–V2 region^8^, giving amplicons with an average length of ≈ 310 bp which is not ideal for the Illumina MiniSeq, Nextseq, or iSeq—all cost-efficient high-throughput DNA sequencing platforms producing only sequences ≤ 300 bases. We therefore designed a new amplification primer set, based on the previously described S-D-Bact-0049-a-S-21^16^ and S-D-Bact-0338-a-A-19^17^ primers giving an average amplicon length of ≈ 260 bp, including cell adaptors and indices suitable for a one-step amplification protocol (Fig. 2).

**Figure 3:**
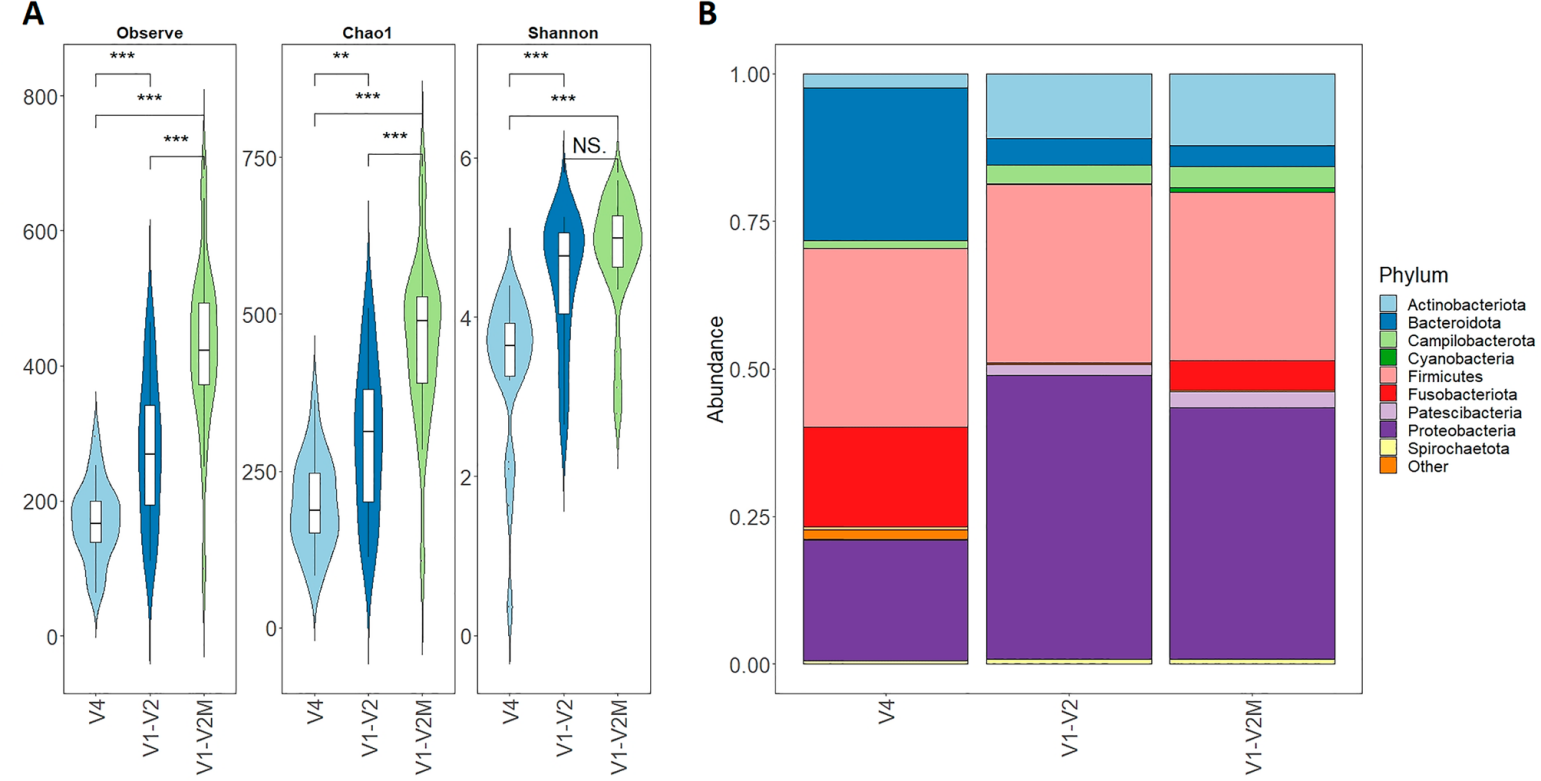
Comparison of esophagus samples using primers targeting V1–V2 and V4 regions of the 16S rRNA gene. (**A**) Comparison of average alpha diversity indices between the samples amplified with V1–V2 and V4 primers. (**B**) Average sample composition at the phylum level—the ten most abundant phyla are shown. Statistical testing was performed using the Wilcoxon test (***< 0.001, **< 0.01, *NS* not significant).

The reanalysis of all biopsy samples from the esophagus with this V1–V2 primer set showed that the number of ASVs aligned to the human genome in all biopsy sites dropped practically to zero (Fig. 1A). Notably, when Walker et al*.* used the S-D-Bact-0027-b-S-20 and S-D-Bact-0338-a-A-18 primers about 30% of the reads still aligned to human DNA^12^. Indeed, the rarefaction curve produced by the sequencing data corresponding to the V1–V2 and V4 primer pairs showed significantly more ASVs in samples amplified with the V1–V2 primers (Supplementary Fig. S1) and these primers consistently also have significantly higher alpha diversity indices compared to primers targeting the V4 region (Fig. 3A) confirming the higher taxonomic resolution that has been observed previously^6^. Analysis of the ten most abundant phyla in both analyzed regions corresponded to the typical bacteria composition of the upper gastrointestinal (GI) tract^18–22^. Pairwise comparison of samples amplified with V1–V2 and V4 primers showed a significantly higher representation of *Actinobacteria* and *Proteobacteria*, a lower representation of phylum *Bacteroidota,* and the absence of the phylum *Fusobacteriota* in samples amplified with V1–V2 primers (Fig. 3B). Similar differences have been observed in recent studies analyzing the bacteriome structure of esophageal biopsies, between the samples analyzed with V4^23,24^ or V3–V4^25,26^ primers when our results from the V1–V2 region are very close to the bacteriome profile of Li et al. analyzed with the V3–V4 primers^26^ from esophagus biopsies. However, due to a total absence in samples amplified with V1–V2 primers of phylum *Fusobacteriota* in the esophageal microbiota^27^, we did an alignment of both V1–V2 primers with the 16S rRNA gene of *Fusobacteriota*. This showed a two-base mismatch at the 3′terminus of the S-D-Bact-0049-a-S-21 primer and thus we designed an extra forward primer 68F_M (Table 1) targeting *Fusobacteriota* and together with the original primers once again amplified all biopsy samples from the esophagus. The community structure in samples amplified with this modified mixture of V1–V2 primers (V1–V2M) showed significantly more observable species thanks to the amplification of phylum *Fusobacteriota* (Fig. 3A), although the profile was generally similar to that obtained using the original V1–V2 primers (Fig. 3B).

**Table 1:** Primers used for 16S rRNA gene sequencing analysis.

16S region	Name	c (µmol L^−1^)	Sequence (5′–3′)
V4	515F^36^	0.2	5′AATGATACGGCGACCACCGAGATCTACACGCTGCGTAAGATATGGTAATTGTGTGYCAGCMGCCGCGGTAA 3′
	806R^36^	0.2	5′CAAGCAGAAGACGGCATACGAGATTCGCCTTAAGTCAGCCAGCCGGACTACNVGGGTWTCTAAT 3′
	Read1		TATGGTAATTGTGTGCCAGCMGCCGCGGTAA
	Read2		AGTCAGTCAGCCGGACTACHVGGGTWTCTAAT
	Index1		ATTAGAWACCCBDGTAGTCCGGCTGACTGACT
	Index2		TTACCGCGGCKGCTGGCACACAATTACCATA
V1–V2	68F^16^	0.2	5′AATGATACGGCGACCACCGAGATCTACACGCTCGTCTAATAGTCAGCCAGCCGTNANACATGCAAGTCGRRSG 3′
	68F_M	0.1	5′AATGATACGGCGACCACCGAGATCTACACGCTCGTCTAATAGTCAGCCAGCCGTAACACATGCAAGTCRACTYGA 3′
	338R^17^	0.3	5′CAAGCAGAAGACGGCATACGAGATTCATGAGCTATGGTAATTAAGCTGCCTCCCGTAGGAGT 3′
	Read1		AGTCAGCCAGCCGTNANACATGCAAGTC
	Read2		TATGGTAATTAAGCTGCCTCCCGTAGGAGT
	Index1		ACTCCTACGGGAGGCAGCTTAATTACCATA
	Index2		GACTTGCATGTNTNACGGCTGGCTGACT

### Taxonomic richness and composition across the V4 and V1–V2 amplicon datasets

**Figure 4:**
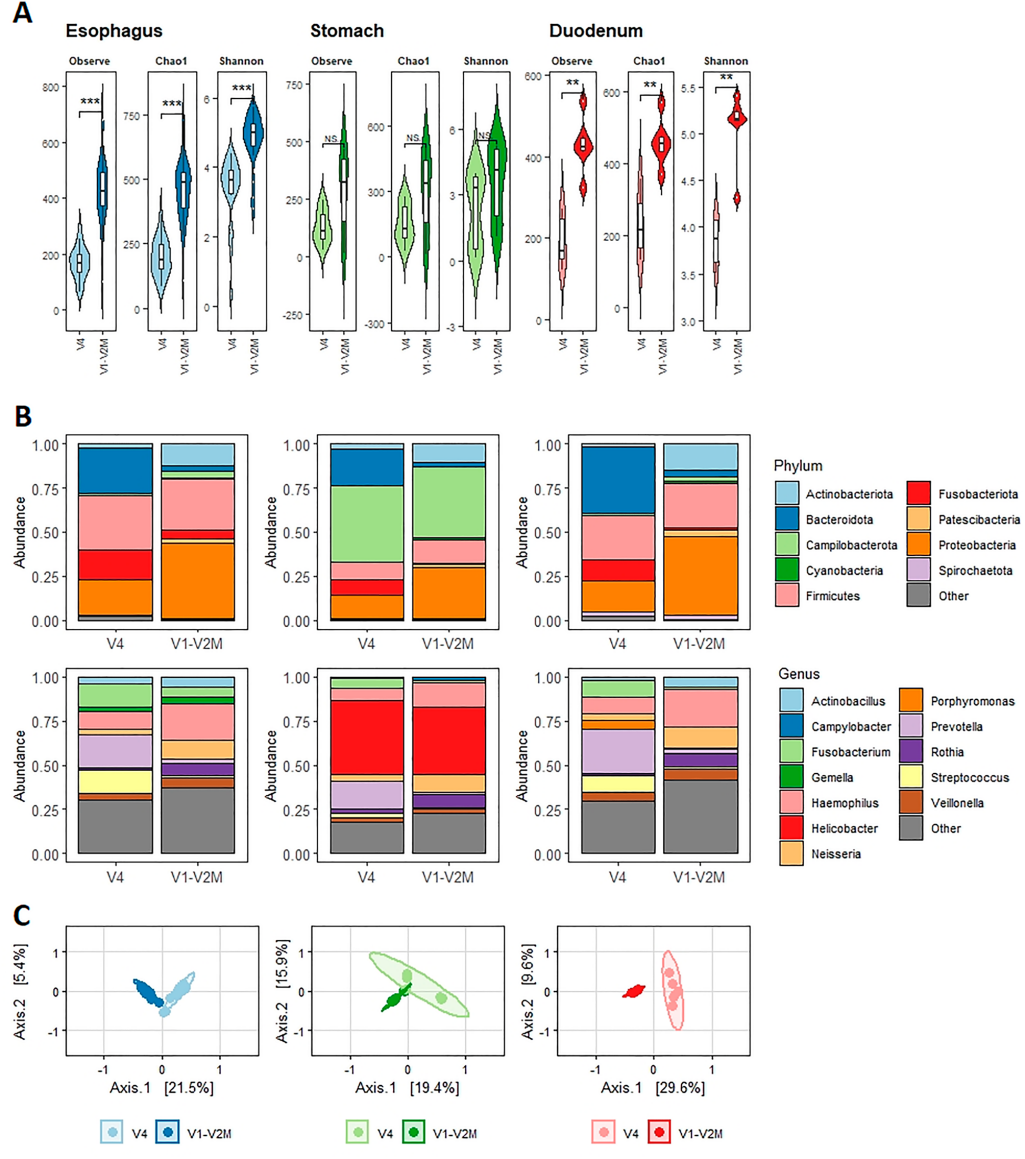
Analysis of biopsy samples from the upper GI tract using primers targeting the V1–V2M and V4 regions of the 16S rRNA gene. (**A**) Comparison of average alpha diversity indices between samples amplified by V1–V2M and V4 primers. Statistical testing was performed using the Wilcoxon test (***< 0.001, **< 0.01, *NS* not significant). (**B**) Average sample composition at the phylum level—the ten most abundant phyla are shown. (**C**) Principal coordinates analysis (PCoA) based on the Jaccard distance; the statistical significance was proved by PERMANOVA. All analyses were done from 6 duodenum biopsies, 11 stomach biopsies, and 23 esophagus biopsies amplified with V1–V2M and V4 primers.

Next, we analyzed the structure of the bacterial community identified using V1–V2M and V4 primers in biopsy samples of the esophagus, stomach, and duodenum representing the complete upper GI tract (Fig. 4). We observed a significantly higher estimated taxonomic richness at the species level in terms of all widely used alpha diversity indices for the esophagus and duodenum samples amplified with V1–V2M primers compared to V4 primers (Fig. 4A). Only gastric biopsy samples showed no observable difference in taxonomic richness between V4 and V1–V2M as a consequence of the high abundance of the *Campylobacterota* phylum (up to 95%, Supplementary Fig. S2) in several patients. This was due to the presence of bacteria *Helicobacter pylori*, the widespread stomach pathogen associated with risk of chronic gastritis, peptic ulcer, and gastric adenocarcinoma^28^. Because the taxonomic composition of the average upper GI tract varied between individual locations (Fig. 4B), we conducted a detailed analysis of taxonomic richness on the biopsy samples from the esophagus as they formed the largest group and were collected from multiple sites in each patient.

**Figure 5:**
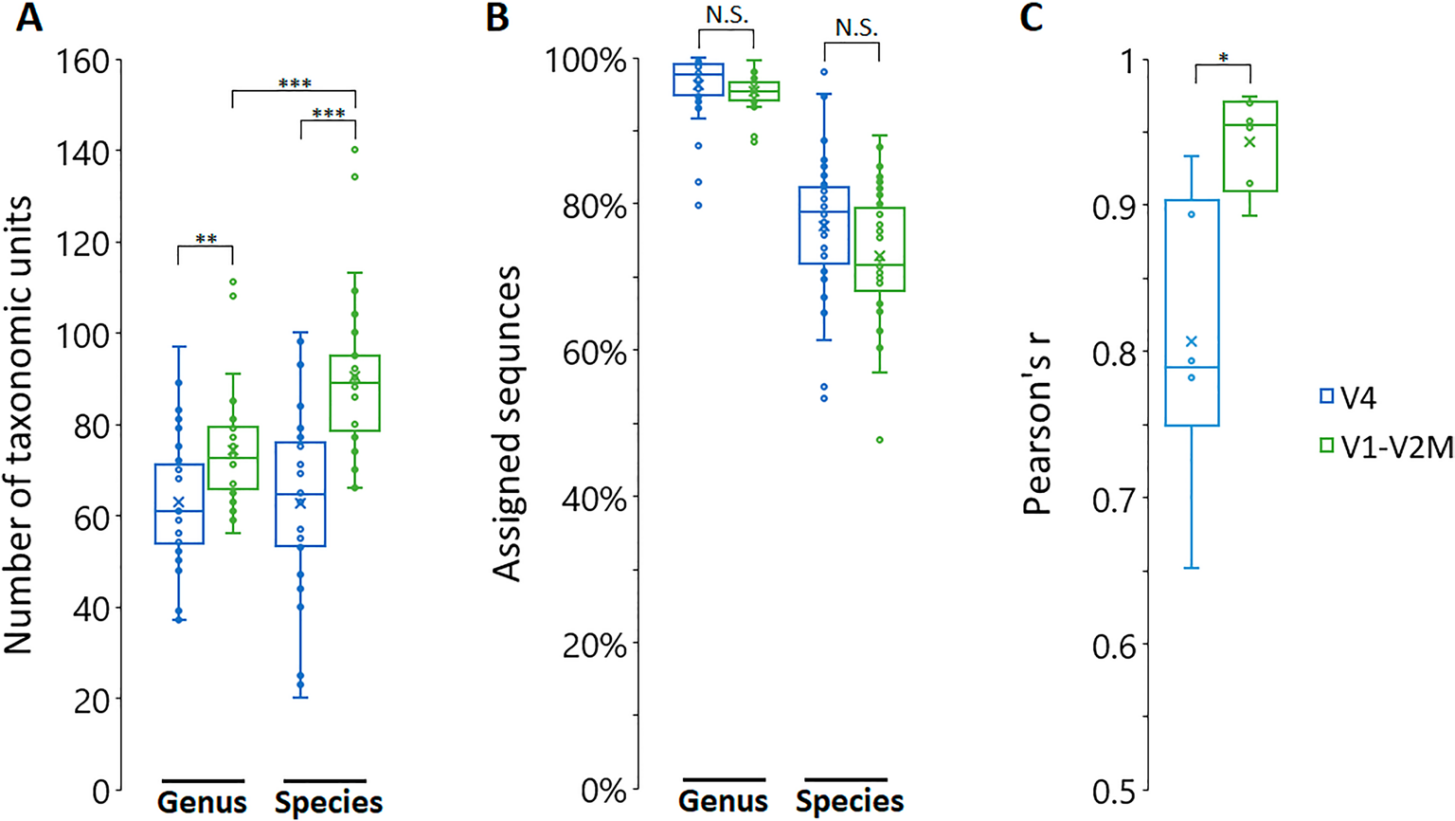
Taxonomic resolution and reproducibility of analysis for each 16S rRNA gene-specific dataset from esophageal biopsy samples. (**A**) Taxonomic richness (genus and species level) with V1–V2M and V4 amplicons (n = 36; 4 samples with a high prevalence of *Helicobacter pylori* were omitted). Statistical testing was performed using the Student’s T-test (***< 0.001, **< 0.01) (**B**) Percentage of sequences with assigned taxonomy (genus and species level) for each amplicon-based dataset (n = 36; 4 samples with a high prevalence of *H. pylori* were omitted). Statistical testing was performed using the Student’s T-test; *NS* not significant (**C**) Pearson’s correlation coefficients calculated from two esophageal biopsy samples collected from one patient for each amplicon-based dataset (n = 6). Statistical testing was performed using the Student’s T-test (*< 0.05).

As expected from rarefaction curve analysis (Supplementary Fig. S1), the V1–V2M primers showed a significantly higher taxonomic richness at the genus and especially at the species level (Fig. 5A). Noticeably, with V4 primers, there was no difference in taxonomic richness between the genus and the species levels. On the other hand, the taxonomic assignment of both primer pairs showed a comparable efficiency on genus and species levels (Fig. 5B). However, when we analyzed the reproducibility of the analysis on six patients we found a significantly higher correlation with V1–V2M primers compared to V4 primers between two esophageal biopsy samples collected from one patient (Fig. 5C).

This low reproducibility suggests PCR bias within the amplification of individual 16S rRNA gene V4 regions as a result of massive off-target amplification. Indeed, this result is in line with sequence entropy (variability) for the V1–V2 and V4 regions^6^ and confirms previous findings from the analysis of urinary and gut microbiota that the V1–V2 16S rRNA gene amplicon is much more informative in terms of taxonomic richness compared to the V4 amplicon^10,11^. On the other hand, analysis of the V1–V2 region did not show poor sequence classification in the identification of bacterial taxa belonging to the phylum *Proteobacteria* which was predicted in a previous in-silico experiment based on 16S rRNA gene sequences from a Greengenes public database^6^.

Regarding phyla with an average representation higher than 0.5% *Bacteroidota*, *Firmicutes*, *Proteobacteria*, *Fusobacteriota*, *Campylobacterota*, *Actinobacteriota* and *Spirochaetota* were detected in both amplicon datasets. *Patescibacteria* and *Cyanobacteria* detected only with the V1–V2M dataset were also present in the V4 dataset but their representation was under 0.1% (Supplementary Fig. S3A). In the case of low abundant phyla (< 0.5%), nine were common to both datasets and some phyla were detected exclusively in a subset with a total average relative abundance of only < 0.01% (Supplementary Fig. S3B). They have been described mainly as thermophilic bacteria or archaea present in soil or hot springs^29–31^ indicating either contamination or taxonomic misclassification. The evaluation of the intersection between genera with an average representation higher than 0.5% present in the V4 and V1–V2M datasets showed that 17 genera were present in both datasets. The second largest group comprised 16 genera present in the V1–V2M dataset and 4 genera present in the V4 dataset. Of the 20 genera present in one dataset, only two (*Capnocytophaga* and *Leptotrichia*) were not identified in the V1–V2M dataset and four (*Cutibacterium, Jeotgalicoccus, Pseudomonas,* and *TM7x*) were not identified in V4 dataset.

**Figure 6:**
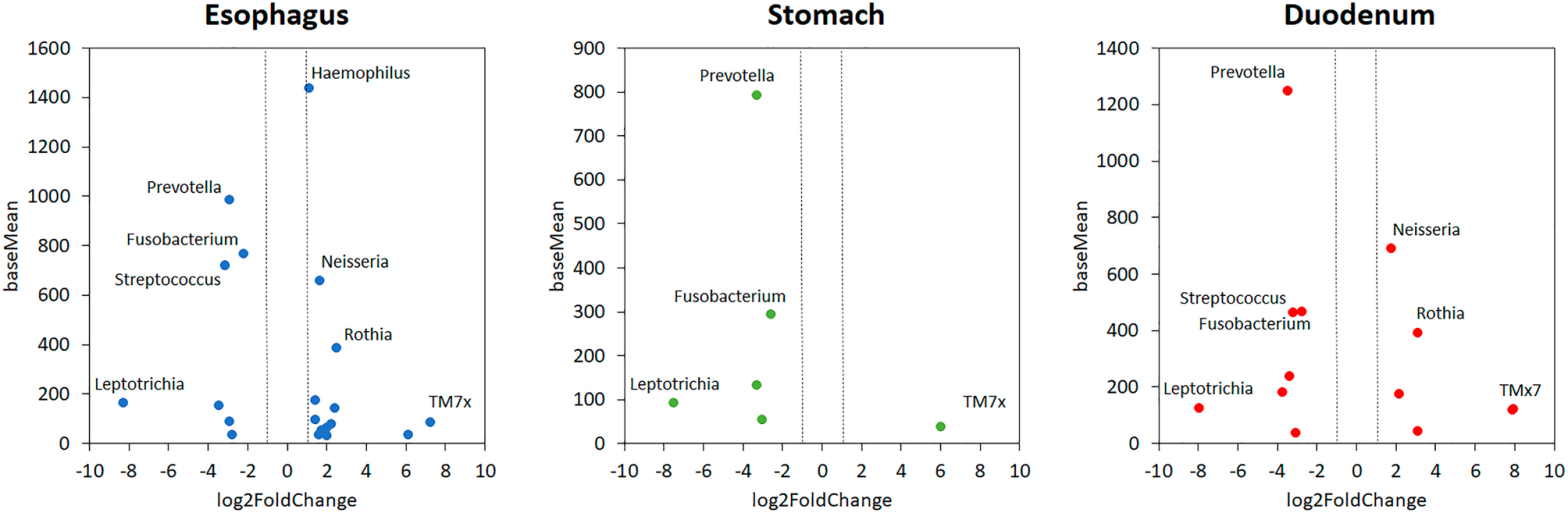
Different genera representations between V1–V2M and V4 amplicon-specific datasets from biopsy samples of the esophagus, stomach, and duodenum. Genera showing statistically different representations (P < 0.05, analyzed by the Wilcoxon test) between amplicons from V1–V2M and V4 regions of the 16S rRNA gene are ranked in the plot according to their abundance (baseMean—Y-axis) and their relative abundance ratio (log2FoldChange—X-axis).

On the other hand, we observed discrepancies between amplicon datasets in bacterial composition by relative taxa abundances. Due to this substantial difference, we performed a beta diversity analysis for each location of the upper GI tract (esophagus, stomach, and duodenum). Using principal coordinates analysis (PCoA) ordered according to the Jaccard distance, we observed statistically significant clustering between V4 and V1–V2M datasets in all locations showing a separation on Axis1 (Fig. 4C). The analysis of significantly different genera (P < 0.05, average presence > 0.5%) between the datasets from the esophagus and duodenum biopsies showed increased representation of the abundant genera *Prevotella, Fusobacterium*, and *Streptococcus* in the V4 dataset and *Neisseria*, *Haemophilus* and *Rothia* in the V1–V2M dataset (Fig. 6). In biopsies from the stomach, we observed increased representation of abundant genera *Prevotella* and *Fusobacterium* only in the V4 dataset (Fig. 6). Indeed, the genus Tmx7 was nearly absent from all V4 datasets, and the genus *Leptotrichia* was nearly absent from V1–V2M datasets (Fig. 6).

The increased representation of the genus *Prevotella* was reflected in the increased representation of the phylum *Bacteriodota* in the V4 dataset and similarly the increased representation of the genera *Neisseria*, *Haemophilus,* and *Rothia* was reflected in the increased representation of the phyla *Proteobacteria and Actinobacteria* in the V1-V2M dataset (Fig. 4B). Based on these results we checked the alignment of both primer sets with all representants showing large discrepancies and we found that there is a 2-base mismatch at the 3′end of S-D-Bact-0049-a-S-21 primer only in the genus *Leptotrichia*, which explains the observed poor amplification of this genus with V1–V2M primers. The differences in relative taxa composition in the two data sets confirm previous studies showing that the particular 16S rRNA gene primer set used substantially influences the analysis of bacteria diversity and composition^10,32–35^. Recent analyses of bacteriomes in biopsies from the upper GI tract using V3–V4 or V4 primer pairs showed that the relative composition of taxa varied widely^23–26^ between the individual primer sets with a higher abundance of *Bacteroidota* in the case of V4 primers^23,24^ and higher abundance of *Actinobacteria* and *Proteobacteria* in the case of V3–V4 primers^25,26^. A similar trend was also described for gut microbiota analysis using V4–V5 and V3–V4 primers^34^. Besides, the higher representation of the genus *Streptococcus* in our V4 dataset is in line with a previous study analyzing oral and mock communities using the Illumina MiSeq platform by using 16S rRNA gene V1–V3 and V3–V4 primers in which authors suggest that the V1–V3 region provided a more accurate representation of oral microbial diversity^35^.

### Mock community analysis

**Figure 7:**
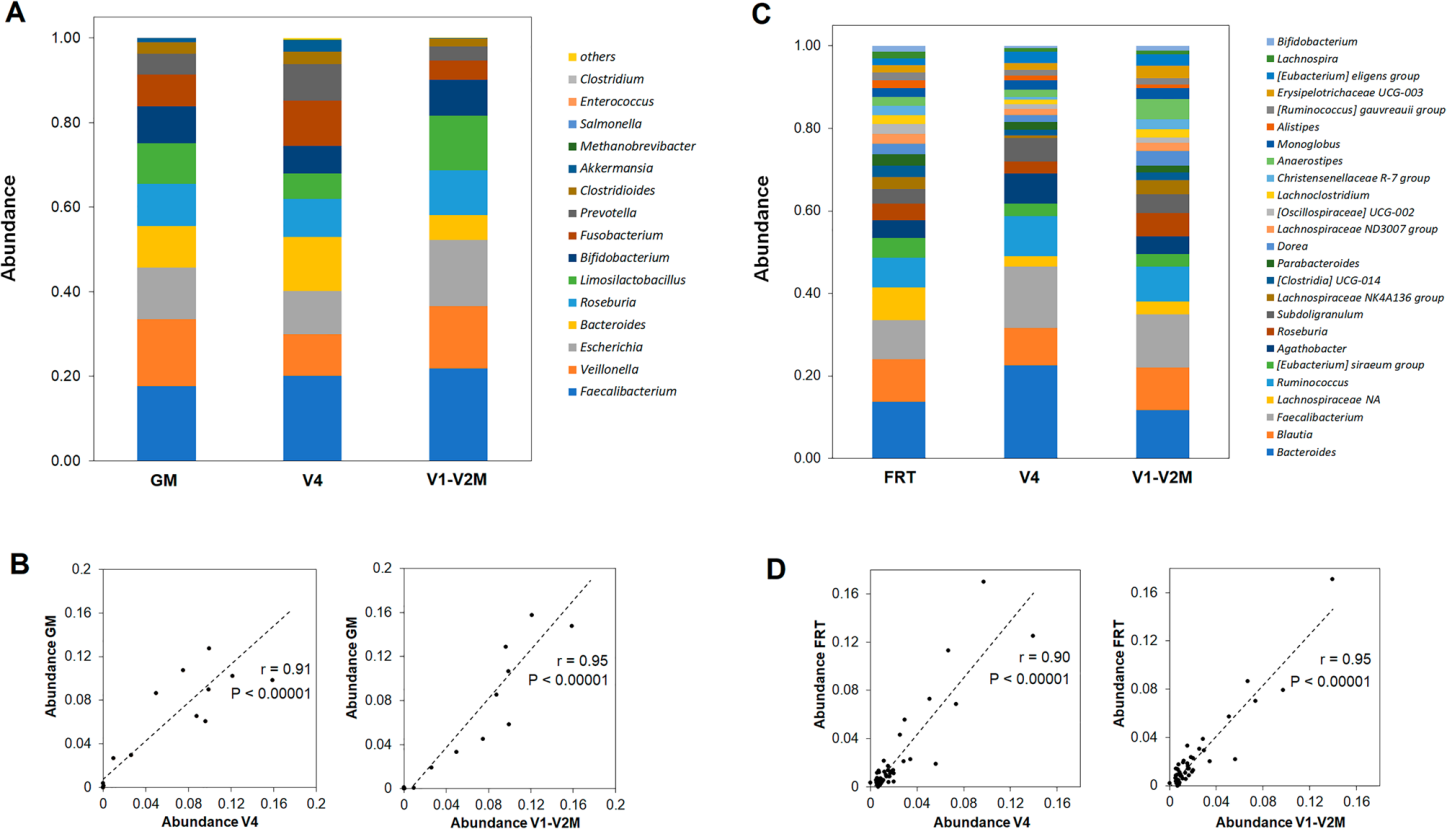
Comparison of the bacterial structure of the ZymoBIOMICS standards analysed with the V4 and V1–V2M primers. ZymoBIOMICS Fecal Reference with TruMatrix Technology (FRT) and ZymoBIOMICS Gut Microbiome (GM) Standard (Zymo Research, USA) analyzed by 16S rRNA gene sequencing using V4 and V1–V2M primer pairs and sequenced on Illumina MiniSeq (2 × 150 bp). (**A**,**C**) Average genus-level sample composition—FRT standard genera representing more than 1% are shown. (**B**,**D**) Scatter plots of the genus abundances for the V4 and V1–V2M primer pairs used for the sequencing of the 16S rRNA gene with the Pearson correlation coefficient.

We sequenced a commercially available mock reference community, ZymoBIOMICS Fecal Reference with TruMatrix Technology (FRT) and ZymoBIOMICS Gut Microbiome (GM) Standard (Zymo Research, USA), to assess bias in V1–V2M primer based microbial composition profiling. The representation of each genus in the GM standard was compared with the data from sequencing the standards using primers V1–V2M and V4. For the FRT standard, available raw sequencing data were analyzed at the level of genera > 1% abundance and compared with our data (Fig. 7). Both data sets, V1–V2M and V4, showed a very high degree of correlation with the bacterial communities in the two standards (Fig. 7B,D). In the case of GM standard, the V4 primers slightly underestimated the genera *Veilonella* and *Limosilactobacillus* and the V1–V2M primers slightly underestimated the genus *Bacteroides*. An analysis of the FRT community showed a slightly higher representation of the genera *Bacteroides*, *Agathobacter* and *Subdoligranulum* in the V4 primer data set and of *Anaerostipes* in the V1–V2M data set. In general, however, these results show that the V1–V2M primers give comparable data to the V4 primers.

The main limitation of this and other studies is usually the lack of information about the true taxonomic composition of the analyzed sample. However, selecting the appropriate 16S rRNA gene hypervariable regions for analysis is a critical consideration for characterizing the relevant bacterial communities and eliminating bias due to off-target amplification. The development of methodology for metagenome analysis of human biopsies is fundamental for both medical research and clinical practice. Currently available 16S rRNA gene sequencing techniques including third-generation sequencing platforms (MinION, PacBio), are not optimal for the examination of low bacterial abundant samples with a predominant ratio of human DNA. In our study, we have designed a set of primers for amplicon V1–V2 16S rRNA gene sequencing of bacteria presented in human biopsies that, in combination with the Illumina MiniSeq/Nextseq/iSeq platforms, maintain the efficiency of this method while at the same time radically reducing its price.

## Methods

### Sample collection

The study was performed with the approval of the Committees for Ethics of the University Hospital in Brno (No. 05-101019/EK, May 15th, 2019). Written informed consent was obtained from all participants before inclusion in the study and the study is in line with the Helsinki declaration. 17 samples from esophagus, 6 samples from esophageal adenocarcinoma, 11 samples from stomach, and 6 samples from duodenum were collected from 7 patients with gastroesophageal reflux disease (GERD) and 6 patients with esophageal adenocarcinoma (EAC) at the Department of Gastroenterology and Internal Medicine, Faculty Hospital Brno. The biopsies were placed in 2 mL sterile tubes with 2 g of 1.4 mm homogenization ceramic beads (Qiagen, Hilden, Germany) and 600 µL RLT lysis buffer from the AllPrep DNA/RNA 96 Kit isolation kit (Qiagen, Hilden, Germany) and frozen immediately at – 80 °C until DNA extraction.

### DNA extraction

Samples were thawed to room temperature and 2-mercaptoethanol (Sigma-Aldrich, St. Louis, Missouri, USA) was added to each sample to a final 1% concentration and mechanically homogenized 2 × 50 s for 6500 RPM using Precellys Evolution homogenizer (Bertin Technologies SAS, France). The samples (and the DNA extraction negative control) were then processed for DNA extraction using the AllPrep DNA/RNA 96 Kit (Qiagen Hilden, Germany) according to the manufacturer’s spin protocol and the eluted DNA was stored at − 20 °C until further analysis. The ZymoBIOMICS Fecal Reference standard (Zymo Research, USA) was processed for DNA extraction using the ZymoBIOMICS DNA Miniprep Kit (Zymo Research, USA) according to the manufacturer’s spin protocol and the eluted DNA was stored at − 20 °C until further analysis.

### 16S rRNA gene sequencing library preparation

Genomic DNA was amplified in a PCR reaction with primers targeting the variable regions V1–V2 (68F^16^-338R^17^) and V4 (515F-806R^36^) of the 16S rRNA gene. The amplification of the V4 region was according to the previously described EMP protocol on MiniSeq and for the V1–V2 region, we added Illumina MiniSeq flow cell adaptors and indices to the previously described primers. The sequences and details of the primers used were processed in OligoAlanyzer (Integrated DNA Technologies, Inc., Coralville, IA, USA) and are provided in Table 1. Amplification of both variable regions was performed in 50 µL reactions, containing 20 µL of Platinum II Hot-Start PCR Master Mix (2X) (Thermo Fisher Scientific, Waltham, USA), 0.1–0.3 µmol L^−1^ of primers (see Table 1) and 6 µL template. The thermal profile started with initial denaturation 94 °C × 3 min, followed by 35 cycles of denaturation at 94 °C × 45 s, annealing at 52 °C × 1 min and extension at 72 °C × 1 min 30 s, and a final extension at 72 °C for 10 min. SPRIselect beads (Beckman Coulter, California, USA) were used for PCR product purification. After verifying the length of the PCR products in the 5200 Fragment Analyzer system (Agilent Technologies, Santa Clara, California, USA) and determining their concentration by the Quantus Fluorometer (Promega, Madison, Wisconsin, USA) the PCR products were pooled at a standardized concentration of 4 nM. The pooled library was prepared and subjected to MiniSeq Mid Output Kit (2 × 150 paired-end sequencing) on the MiniSeq sequencer (Illumina, San Diego, California, USA) using custom sequencing primers for V4 and V1–V2 (see Table 1).

### Negative control

Negative controls consisted of reagent-only controls consisted of empty collection tubes to which all DNA extraction, PCR, and library preparation were added. Three reagent controls were included for each variable region analysis plate.

### 16S rRNA gene sequence analysis

Raw fastq reads were mapped to the human genome hg38 using the Rbowtie2 package (version 1.14.0)^37^. Successfully mapped reads were then subtracted from the dataset. The rest of the reads were processed using the DADA2^38^ package (version 1.20.0) in R (version 4.1.1). The analysis was carried out according to the standard operating procedure with the addition of reads concatenation. Briefly, reads were first filtered and trimmed (maximum of 0 ambiguous bases, expected error threshold of 2 and the last 10 bases truncated). Filtered reads were then de-replicated (unique sequences were extracted) and de-noised (identified sequencing errors were removed using learned error rates and quality profiles of reads). Overlapping reads were merged and non-overlapping reads were concatenated. Chimaeras were then removed, and taxonomy was assigned by the RDP naive Bayesian classifier method^39^ against the SILVA reference database^40^ (version 138.1). The identification and removing of contaminant DNA sequences was done by R package decontam^41^ using widely reproduced signatures of contaminant DNA (Supplementary Table S1). A phylogenetic tree was built using the phangorn^42^ package (version 2.7.1) with the DECIPHER package (version 2.20.0) used for multiple alignments. The phyloseq^43^ (version 1.36.0), vegan^44^ (version 2.6.2), microbiome^45^ (version 1.14.0), MicrobiotaProcess^46^ (version 1.4.4) and DESeq2^47^ (version 1.32.0) packages were used for subsequent phylogenetic and statistical analyses, and the packages ggplot2, ggtree^48^ (version 3.0.4) and patchwork^49^ (1.1.1) was used for producing graphical outputs.

### Ethics declarations

The study was performed with the approval of the Committees for Ethics of the University Hospital in Brno (No. 05-101019/EK, May 15th, 2019). Written informed consent was obtained from all participants before inclusion in the study and the study is in line with the Helsinki declaration.

### Supplementary Information

Supplementary Information 1.

Supplementary Information 2.

Supplementary Information 3.

## Data Availability

Datasets generated and analyzed during the current study are available in the SRA under BioProject IDs: PRJNA877810 and PRJNA995527. The DADA2 codes used to analyze the data are provided in the supplementary material.
