# Supplementary material for: 16S rRNA gene primer choice impacts off-target amplification in human gastrointestinal tract biopsies and microbiome profiling
Source: Sci Rep. 2023 Aug 3;13:12577. doi: 10.1038/s41598-023-39575-8 (PMC10400661; doi:10.1038/s41598-023-39575-8)
Supplement: Supplementary file 2 — Supplementary Information 2. [file 41598_2023_39575_MOESM2_ESM.docx]

**Supplementary Figures**

**16S rRNA gene primer choice impacts off-target amplification in human gastrointestinal tract biopsies and microbiome profiling**

Tereza Deissová, Martina Zapletalová, Lumír Kunovský, Radek Kroupa, Tomáš Grolich, Zdeněk Kala, Petra Bořilová Linhartová and Jan Lochman


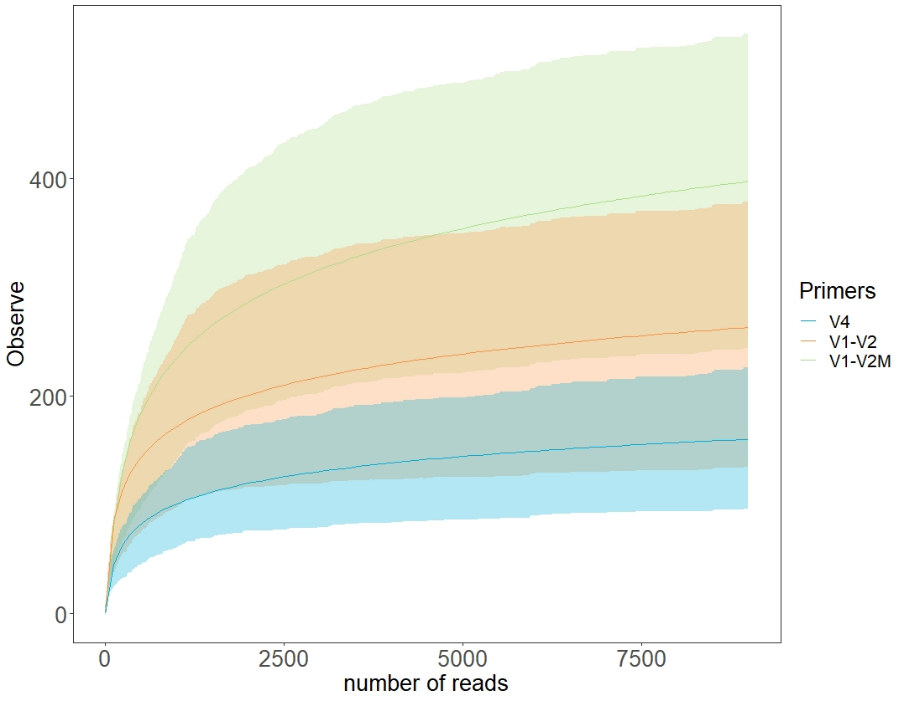


**Figure S1.** Rarefaction curves of samples as functions of observed ASVs (amplicon sequence variants) produced by the sequencing data corresponding to V1-V2, V1-V2M, and V4 primer sets. The analysis was done in vegan^42^ (version 2.6.2)


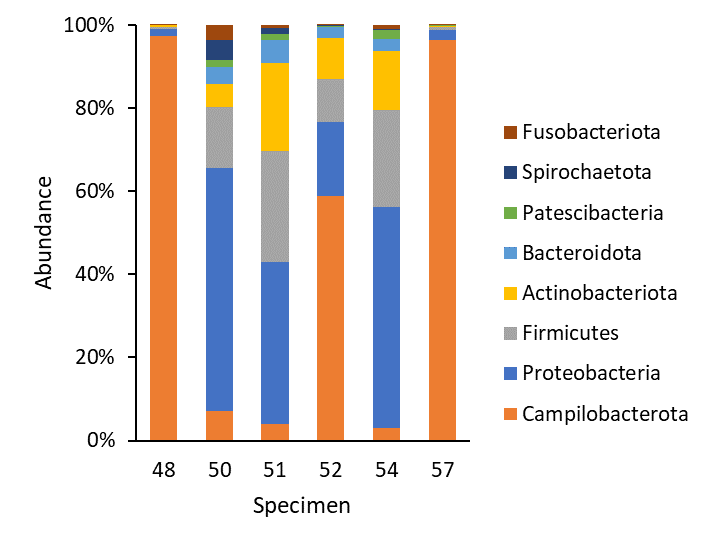


**Figure S2.** Sample composition of selected gastric biopsies at the phylum level using primers targeting the V1-V2 region of 16S rRNA gene – the eight most abundant phyla are shown.


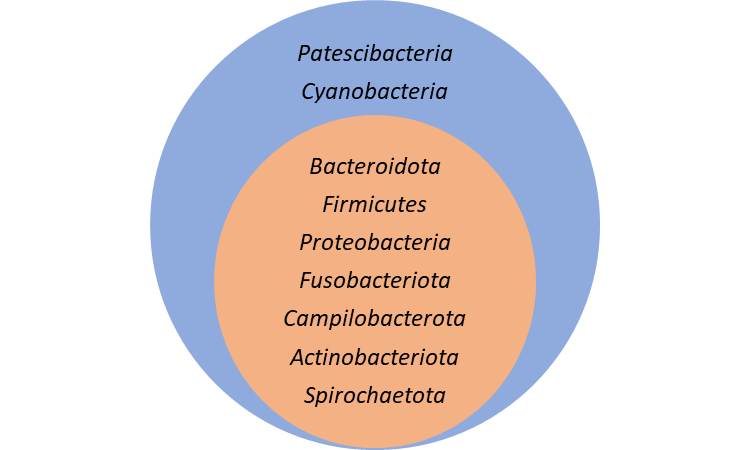


**A**

**B**


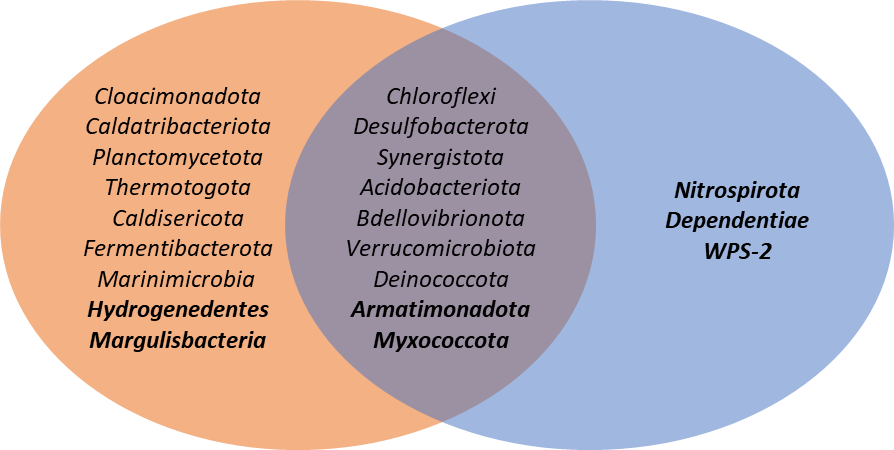


**V4**

**V1-2M**

**V1-2M**

**V4**

**Figure S3.** The presence of phyla in biopsy samples from the upper GI was analyzed by V4 and modified V1-V2 primer sets. (A) Phyla with an average representation higher than 0.5%. (B) Phyla with an average representation lower than 0.5%, the phyla with a total of <0.01% average relative abundance are shown in bold.
